# Research on basis of reverse genetics system of a Sindbis-like virus XJ-160

**DOI:** 10.1186/1743-422X-8-519

**Published:** 2011-11-14

**Authors:** Zhu Wu-yang, Liang Guo-dong

**Affiliations:** 1 State Key Laboratory for Infectious Disease Prevention and Control, National Institute for Viral Disease Control and Prevention, Chinese Center for Viral Disease Control and Prevention, Beijing 100052, China

## Abstract

As a Sindbis-like virus (SINLV), XJ-160 virus was isolated from a pooled sample of *Anopheles *mosquitoes collected in Xinjiang, China, in 1990. Recombinant plasmid pBR-XJ160 is an infectious full-length cDNA clone of XJ-160 virus, from which rescued virus BR-XJ160 can be obtained by transcription in vitro and transfection. The BR-XJ160 virus raised in BHK-21 cells was indistinguishable from the XJ-160 virus in its biological properties, including its plaque morphology, growth kinetics and suckling mouse neurovirulence. On basis of pBR-XJ160, the effects of substitutions within nonstructural protein 1 (nsP1) or nsP2 on the infectivity and pathogenesis of Sindbis virus (SINV) have been investigated. We have also confirmed the essential role of E2 glycoprotein, especially the domain of 145-150 (amino acid) aa, in SINV infection through the interaction with cellular heparan sulfate (HS). In addition, we have developed XJ-160 virus-based vector system, including replicon vector, defective helper (DH) plasmids and the packaging cell lines (PCLs). Here we provide an update of main development in the field concerned with XJ-160 virus.

## Introduction

Sindbis viruses are enveloped, single-strand RNA viruses, belonging to the genus of *Alphavirus *in the family *Togaviridae *that has more than 30 members [1]. Some of the members of the *Alphavirus *genus, such as Venezuelan, Eastern, and Western equine encephalitis viruses (VEEV, EEEV, and WEEV, respectively), can cause fever and viral encephalitis in human beings, resulting in mass epidemics or outbreaks exampled by the occurrences in South America and North America [2-4]. Due to the relative difficulty and risk of handling these dangerous pathogenic alphaviruses, Sindbis virus (SINV), that normally causes a mild rash and arthritis in humans but can cause fatal encephalomyelitis in mice, has been used extensively as a model system for the study of the infectivity and pathogenesis of alphaviruses.

Sindbis virus genome is a single strand of positive-sense RNA of approximately 12 kb which is capped at the 5' terminus and polyadenylated at the 3' terminus. The 5' two-third of this RNA encodes the nonstructural proteins (nsP1 through 4). The 3' one-third is initially translated as a polyprotein (NH2-C-E3-E2-6K-E1-COOH) that is processed posttranslationally to produce the structural proteins (SPs) (capsid, El and E2). In infected cells, the virion structural proteins are translated from a subgenomic mRNA (26S RNA) and produced by transcription of genome-length complementary (minus) strand from a highly active subgenomic promoter [5]. Based on the divergence of nucleotide sequencing and biological characteristics, Sindbis virus can be divided into two groups: SINV and Sindbis-like virus (SINLV) [5]. XJ-160 virus (GenBank No. AF103728) was isolated from a pool of Anopheles mosquitoes collected in Xinjiang, China [6]. Similar to other SINVs, XJ-160 virus causes fever, rash, and arthritis in humans but causes an age-dependent encephalitis in mice. Neonatal (2 days old) mice injected intracerebrally with XJ-160 died within 48 h; however, intracerebral inoculation of XJ-160 was not lethal to 3-week-old mice. The overall genomic sequence of XJ-160 has diverged significantly from the prototype AR339 with an 18% difference in nucleotides and an 8.6% difference in aa sequence. Therefore, XJ-160 was accordingly characterized as a SINLV [6]. In this review, we will provide an overall summary on the progress acquired in the field concerned with XJ-160 virus.

### Isolation and complete nucleotide sequence of XJ-160 virus

Infection with alphaviruses is common in the Chinese population. In 1990, we have reported the isolation of a Sindbis-like virus from a pool of *Anopheles *mosquitoes collected in Xinjiang, China during an arbovirus survey. This virus, designated XJ-160, rapidly produced cytopathic effects (CPE) on mosquito and hamster cells [6]. Biological and serological studies show this virus is a new subtype of Sindbis virus firstly isolated in China. Complete sequence of the genomic RNA of XJ-160 virus contains 11626nt and shows typical genomic organization of Sindbis virus. However, nucleotide difference between XJ-160 virus and prototype Sindbis virus (AR339 strain) is 18%, much more than the nucleotide difference (2~7%) among the Sindbis virus. In addition, XJ-160 virus possesses some special biological characteristics, for example, we found that XJ-160 virus can be neutralized by anti-Sindbis antibody, but SINV can't be neutralized by anti-XJ-160 antibody, i.e. it has nonreciprocal serological reaction between XJ-160 virus and SINV. In addition, there are 11 deletions and 2 insertions, involving 99 nucleotides in total. XJ-160 is most closely linked to Kyzylagach virus isolated in Azerbaijan. Both belong to the African/European genetic lineage of Sindbis virus, albeit more distantly related to other members [6]. These results indicate that there are some differences about structure and function between XJ-160 virus and SINV, and XJ-160 virus is a new subtype of Sindbis virus.

### Construction of infectious clone for XJ-160 virus

Research investigating positive-sense RNA viruses has been considerably advanced by the development of the reverse genetics system. Recombinant cDNA clones from which full-length infectious RNA can be transcribed are a valuable tool for studying the molecular biology of positive-strand RNA viruses [7]. The approach relies on the infectious nature of the genome RNA of these viruses when transfected into permissive host cells. To construct the reverse genetics system, the full-length cDNA of XJ-160 virus was amplified from viral RNA and cloned into plasmid derived from pBR322 under the control of a SP6 promoter and followed by a polyA tail behind 3'terminus of genomic cDNA. RNA transcribed from the full-length cDNA clone was highly infectious, after transfection into BHK-21 cell, resulting in generation of recovered virus with titer of 10^7^-10^8^PFU/ml [8]. A silent nucleotide change was introduced into the cDNA clone to create a *Xba *I site (C to T at 8453nt) that is used as a genetic marker of this infectious clone. This genetic marker was retained in the genome of recovered virus. The recovered virus was indistinguishable from the parental virus XJ2160 in the aspect s of cytopathogenic effect, plaque morphology on BHK-21cells, viral antigenicity, *in vitro *growth characteristics in BHK-21 cells and the virulence in suckling mice [8]. The stable infectious cDNA clone of XJ-160 strain, as an effective reverse genetics system, will provide a valuable molecular biological tool to study the pathogenesis and replication of Sindbis virus and to develop the vector system of alphaviruses.

### Effects of substitutions within nsP1 or nsP2 gene on characteristics of SINV

During the process of constructing infectious clone for XJ-160 virus, two nucleotide mutations within nonstructural protein 1 (nsP1) appeared to be highly associated with the infectivity of in-vitro synthesized RNA, which led to predicted amino acid changes at residue 169 from Lys to Arg, and at residue 173 from Thr to Ile. The nsP1 region is thought to be involved in minus-strand synthesis [9] and appears to be associated with methyltransferase activity and viral RNA capping [10-13]. However, it is not clear that whether both or one of the two mutations in nsP1 cause lethality of the initial cDNA construct, or what is the effect of substitution on biological characteristics of XJ-160 virus. To assess the relative contribution of nsP1-169Lys or nsP1-173Thr to the infectivity and the characteristics of XJ-160 virus, we used site-directed mutagenesis to obtain clones encoding a change to Arg at residue 169 of nsP1 (pBR-169), a change to Ile at residue 173 (pBR-173), or both changes (pBR-6973) [14]. While the infectivity of in-vitro synthesized RNA from pBR-169 was completely abolished, the rescued viruses BR-173 and BR-6973 could be obtained from pBR-173 and pBR-6973 respectively. Further, compared with BR-XJ160 virus without any mutation, BR-173 virus exhibited higher propagation capacity in cell culture and enhanced neurovirulence in sucking mouse model. Simultaneously, BR-6973 virus with double mutations possessed a medium phenotype in both growth properties and sucking mouse neurovirulence. In addition, to different extent, both BR-173 virus and BR-6973 virus expressed an increased sensitivity to 3-Deazaadenosine (3-DZA), an adenosine analog which is an inhibitor of S-adenosylhonocysteine hydrolase [14]. These results establish that the mutagenesis at residue 169 in nsP1 of XJ-160 virus is a lethal mutation, but the mutation at residue 173 from Thr to Ile can not only enhances viral infectivity, growth capacity and neurovirulence, also compensates the damage to viral viability brought by the mutation at the residue 169. Moreover, these functional changes caused by these two mutations maybe associated with the viral RNA methyltransferase activity of nsP1 of XJ-160 virus.

As one of the nonstructural proteins, nsP2 is a component of the replicative enzyme complex required for replication and transcription of viral RNAs but also plays a role in suppressing the antiviral response in infected cells. Mutations in nsP2 of SINV, especially point mutations at nsP2-726 Pro, are often responsible for persistent infection or prolonged survival of infected cells [15-17] and have been used to construct alphavirus-based replicons with reduced cytotoxicity [18,19]. However, the effects of nsP2-P726 mutation on characteristics of Sindbis virus in the context of genomic-RNA are poorly understood. To investigate the effect of point mutation at nsP2-P726 on characteristics of Sindbis virus, based on the infectious clone pBR-XJ160, we constructed mutant clone pBR-726L, pBR-726S, pBR-726V and pBR-726A respectively containing a point mutation Pro-to-Leu, Pro-to-Ser, Pro-to-Val and Pro-to-Ala at position 726 of nsP2 [20]. Mutant virus BR-726A virus or BR-726V virus with Ala or Val substitutions exhibited growth characteristics similar to that of BR-XJ160 virus from wild-type infectious clone in cultured cells, including the process of CPE, plaque morphology and growth kinetics. In the case of Leu substitution, no CPE and plaques could be seen after 6 passages through BHK-21 cells, although expression of XJ-160 virus-specific protein could be constantly detected by indirect immunofluorescence assay (IFA) [20]. For the Ser substitutions, an intermediate phenotype was observed. To our surprise, the mutant viruses exhibited the neurovirulence mismatching their propagation capacity either in cultured cells or in mouse brain. Compared with the neurovirulence of BR-XJ160 virus, conversion of Pro to Ala at nsP2-726 highly increased viral neurovirulence, while BR-726V virus with Val substitution exhibited a remarkably attenuated phenotype, although both BR-726A virus and BR-726V virus grew as well as or better than did the wild-type BR-XJ160 virus in tissue culture and their titers in the brains of mice inoculated were similar to that of BR-XJ160 virus [20]. Consistent with viral growth properties is that BR-726S possessed an intermediate neurovirulence phenotype. In addition, BR-726L virus lost completely lethality to the sucking mouse inoculated, even couldn't cause 3-day-old mouse morbidity, including weight loss and hind limb paresis. These results indicate that the residue of nsP2-726 in Sindbis virus not only regulates directly virus-host cell interactions, but also plays an important role in the viral neurovirulence in suckling mouse model.

### The major role of E2 glycoprotein in the nonreciprocal neutralizing reaction between SINV and SINLV

As a SINLV, XJ-160 virus is efficiently neutralized by Sindbis-specific antisera; however, Sindbis virus strain YN87448 which was isolated from human serum sample in China is poorly neutralized by antisera raised to XJ-160 virus [6,21]. To determine the molecular basis for this nonreciprocal neutralization phenotypes between them, we constructed three recombinant XJ-160/SIN viruses designated XJ160/SINE1, XJ160/SINE2 and XJ160/SINE1E2, in which the glycoprotein E1 and E2 whole sequences of XJ-160 virus was replaced separately or simultaneously by that of SINV [22]. Recombinant viruses caused a rapid appearance of CPE, same as the SINV in BHK-21cells. Control cells infected with XJ-160 virus did not show CPE until 36 to 40 h after infection. For recombinant viruses, plaque-forming time was shorter and the size of plaque larger than XJ-160 virus. XJ160/SINE1E2 replicated efficient than that of XJ160/SINE1 and XJ160/SINE2 [23]. In addition, the substitution of E1 or E2 glycoprotein does not alter the virulence property of virus in adult mice. Both recombinant viruses and parental viruses were fatal for suckling mice. XJ160/SINE1E2 and XJ160/SINE2 showed higher neurovirulence than XJ-160 virus and SINV respectively [23]. More importantly, neutralizing antibody of XJ-160 virus can neutralize XJ160/SINE1, but not for XJ160/SINE2 or XJ160/SINE1E2 which replaced the E2 gene of SINV, suggesting that the substitutions of E gene of SINV for that of XJ-160 virus have no remarkable effects on assembly and budding in BHK-21 cells as well as neurovirulence in adult mice, but E2 glycoprotein of SINV plays a major role in the neurovirulence in suckling mice and nonreciprocal neutralizing reaction between XJ-160 virus and SINV.

### The crucial role of E2 glycoprotein interacting with HS in SINV infection

The cell surface receptors for SINV to infect a broad variety of species have not yet been conclusively determined, but it has recently been shown that cell surface heparan sulfate (HS) found in both vertebrate and invertebrate species is involved in SINV infection and pathogenisis [24,25]. Cell culture-adapted strains of SINV initially attach to cells by the ability to interact with HS through selective mutation for positively charged amino acid (aa) scattered in E2 glycoprotein [24-27]. Here we have further confirmed that interaction of E2 protein with HS is crucial for cellular infection of SINV based on the reverse genetic system of XJ-160 virus. Both SINV YN87448 and SINLV XJ-160 displayed similar infectivity on BHK-21, Vero, or C6/36 cells, but XJ-160 failed to infect mouse embryonic fibroblast (MEF) cells [6]. The molecular mechanisms underlying the selective infectivity of XJ-160 were approached by substituting the E1, E2, or both genes of XJ-160 with that of YN87448, and the chimeric virus was denominated as XJ-160/E1, XJ-160/E2, or XJ-160/E1E2, respectively. In contrast to the parental XJ-160, all chimeric viruses became infectious to wild-type MEF cells (MEF-*wt*). While MEF-Ext^-/- ^cells, producing shortened HS chains, were resistant not only to XJ-160, but also to YN87448 as well as the chimeric viruses, indicating that the inability of XJ-160 to infect MEF-*wt *cells likely due to its incompetent discrimination of cellular HS [28]. Treatment with heparin or HS-degrading enzyme resulted in a substantial decrease in plaque formation by YN87448, XJ-160/E2, and XJ-160/E1E2, but had marginal effect on XJ-160 and XJ-160/E1, suggesting that E2 glycoprotein from YN87448 plays a more important role than does E1 in mediating cellular HS-related cell infection [28]. In addition, the peptide containing 145-150 aa from E2 gene of YN87448 specifically bound to heparin, while the corresponding peptide from the E2 gene of XJ-160 essentially showed no binding to heparin [29]. As a new dataset, these results clearly confirm an essential role of E2 glycoprotein, especially the domain of 145-150 aa, in SINV cellular infection through the interaction with HS. Accordingly, SINV is proposed to be divided into HS-dependent group and HS-independent group based on the structure of E2 protein.

### Construction and application of XJ-160 virus-derived vector

Alphaviruses are positive-strand RNA viruses that can mediate efficent cytoplasmic gene expression in insect and vertebrate cells. The concept that alphaviruses can be developed as expression vectors was first established by Xiong et al. [30]. To construct vector system of XJ-160 virus, recombinant vector together with its helper plasmid pBR-H were derived from XJ-160 viral infectious clone pBR-XJ160 by overlap-PCR [31,32]. The quantitative and qualitative results indicate that the replicon vector system is capable of self-replicating in host cell, and the expressing efficiency of heterologous genes is correspondent with that of the commercial Sindbis vector (pSinRep5). The conventional approaches producing infectious Sindbis virus RNA and its derived complementary vectors were restricted primarily to in vitro transcription of cDNA clones from a bacteriophage RNA polymerase promoter, followed by transfection into permissive cells. Compared with this method, there is no need for in vitro transcription and mRNA capping for DNA-based vectors. We have constructed XJ-160 virus-based DNA vector by placing its recombinant genome under the control of the human cytomegalovirus (CMV) promoter of the plasmid pVAX1 (Invitrogen, USA), in which viral structure genes were replaced by the sequence of multiple cloning site (MCS) to allow for insertion of heterologous genes [33]. And a packaging cell lines (PCLs) BHK-21^E+Capsid ^for XJ-160 virus was constructed by two times of selections with G418 and Hygromycin., which not only highly increased packaging efficiency of the replicon vector from XJ-160 virus, but also provided packaging function for the replicon vector from Semliki Forest virus [34].

Based on XJ-160 virus-derived vector pVaXJ, we have establised a defective Sindbis virus replicon-based detection method which could identify Alphaviruses in the cultivation process of unknown viruses. The qualitative or quantitative result shows that this method can be used to identify and detect a variety of Alphaviruses, while it does not react to flaviviruses and bunyaviruses which are also positive-strand RNA viruses. Sensitivity test results show that 1PFU of Alphaviruses can be detected using this method [unpublished]. The detection method is simple, rapid and specific, being expected to be used in clinical examination and epidemiological surveillance as well as rapid screening of viral biological warfare agents.

### Summary

As a SINLV, XJ-160 virus was isolated from a pooled sample of *Anopheles *mosquitoes collected in Xinjiang in 1990. Then its reverse system was constructed, on which we have performed a series of research work, including the effects of mutagenesis of nsPs on biological characteristics of SINV, the role of E2 glycoprotein in the nonreciprocal interaction between SINLV and SINV as well as the function of E2 glycoprotein interacting with HS in the cellular infection. We have constructed RNA- or DNA-based vector derived from XJ-160, and the PCLs for XJ-160 virus was also selected. Currently, we have labeled XJ-160 virus by inserting the reporter gene (EGFP or GLUC) into the genome. The labeled viruses were used to research on the cell tropism of SINV in detail. We first found that SINV could infection renal carcinoma cell persistently in large range, and this discovery indicate that SINV have the potential to be used in kidney cancer treatment.

## Competing interests

The authors declare that they have no competing interests.

## Authors' contributions

WZ collected the references concerned with this review and drafted the manuscript, and GL also contributed to edit and revise the manuscript. Both authors read and approved the final manuscript.

## References

[B1] Griffin DE Alphavirus Fields Virology 2001 4 917 962

[B2] Deardorff ER Forrester NL Travassos-da-Rosa AP Estrada-Franco JG Navarro-Lopez R Tesh RB Weaver SC Experimental infection of potential reservoir hosts with Venezuelan equine encephalitis virus, Mexico Emerg Infect Dis 2009 15 519 525 10.3201/eid1504.081008 19331726 PMC2671456

[B3] Young DS Kramer LD Maffei JG Dusek RJ Backenson PB Mores CN Bernard KA Ebel GD Molecular epidemiology of eastern equine encephalitis virus, New York Emerg Infect Dis 2008 14 454 460 10.3201/eid1403.070816 18325261 PMC2570827

[B4] Weaver SC Ferro C Barrera R Boshell J Navarro JC Venezuelan equine encephalitis Annu Rev Entomol 2004 49 141 174 10.1146/annurev.ento.49.061802.123422 14651460

[B5] Strauss JH Strauss EG The alphaviruses: gene expression, replication, and evolution Microbiol Rev 1994 58 491 562 7968923 10.1128/mr.58.3.491-562.1994 PMC372977

[B6] Liang GD Li L Zhou GL Fu SH Li QP Li FS He HH Jin Q He Y Chen BQ Hou YD Isolation and complete nucleotide sequence of a Chinese Sindbis-like virus J Gen Virol 2000 81 1347 1351 10769078 10.1099/0022-1317-81-5-1347

[B7] Boyer JC Haenni AL Infectious transcripts and cDNA clones of RNA viruses Virology 1994 198 415 426 10.1006/viro.1994.1053 8291226

[B8] Yang YL Liang GD Fu SH Liang GD The construction of replicon of XJ-160 virus Virol Sin 2003 18 221 226

[B9] Wang YF Sawicki SG Sawicki DL Sindbis virus nsP1 functions in negative-strand RNA synthesis J Virol 1991 65 985 988 1824787 10.1128/jvi.65.2.985-988.1991 PMC239844

[B10] Mi S Durbin R Huang HV Rice CM Stollar V Association of the Sindbis virus RNA methyltransferase activity with the nonstructural protein nsP1 Virology 1989 170 385 391 10.1016/0042-6822(89)90429-7 2728344

[B11] Mi S Stollar V Both amino acid changes in nsP1 of Sindbis virusLM21 contribute to and are required for efficient expression of the mutant phenotype Virology 1990 178 429 434 10.1016/0042-6822(90)90340-W 2219702

[B12] Mi S Stollar V Expression of Sindbis virus nsP1 and methyltransferase activity in Escherichia coli Virology 1991 184 423 427 10.1016/0042-6822(91)90862-6 1831311

[B13] Wang HL O'Rear J Stollar V Mutagenesis of the Sindbis virus nsP1 protein: effects on methyltransferase activity and viral infectivity Virology 1996 217 527 531 10.1006/viro.1996.0147 8610444

[B14] Zhu WY Yang YL Fu SH Wang LH Zhai YG Tang Q Liang GD Substitutions of 169Lys and 173Thr in nonstructural protein 1 influence the infectivity and pathogenicity of XJ-160 virus Arch Virol 2009 154 245 253 10.1007/s00705-008-0298-0 19118404

[B15] Dryga SA Dryga OA Schlesinger S Identification of mutations in a Sindbis virus variant able to establish persistent infection in BHK cells: the importance of a mutation in the nsP2 gene Virology 1997 228 74 83 10.1006/viro.1996.8364 9024811

[B16] Frolov I Hoffman TA Pragai BM Dryga SA Huang HV Schlesinger S Rice CM Alphavirus-based expression vectors: strategies and applications Proc Natl Acad Sci USA 1996 93 11371 11377 10.1073/pnas.93.21.11371 8876142 PMC38064

[B17] Perri S Driver DA Gardner JP Sherrill S Belli BA Dubensky TW Jr Polo JM Replicon vectors derived from Sindbis virus and Semliki forest virus that establish persistent replication in host cells J Virol 2000 74 9802 9807 10.1128/JVI.74.20.9802-9807.2000 11000258 PMC112418

[B18] Agapov EV Frolov I Lindenbach BD Prágai BM Schlesinger S Rice CM Noncytopathic Sindbis virus RNA vectors for heterologous gene expression Proc Natl Acad Sci USA 1998 95 12989 12994 10.1073/pnas.95.22.12989 9789028 PMC23682

[B19] Lundstrom K Abenavoli A Malgaroli A Ehrengruber MU Novel Semliki Forest virus vectors with reduced cytotoxicity and temperature sensitivity for long-term enhancement of transgene expression Mol Ther 2003 7 202 209 10.1016/S1525-0016(02)00056-4 12597908

[B20] Zhu WY Fu SH Wang JL He Y Tang Q Liang GD Effects of the nsP2-726 Pro mutation on infectivity and pathogenesis of Sindbis virus derived from a full-length infectious cDNA clone Virus Res 2009 14 204 207 10.1016/j.virusres.2009.01.017 19428754

[B21] Zhou GL Liang GD Li L Fu SH Zhang HL Sequencing analysis of the genome of YN87448 virus, first isolated in China Zhonghua Shi Yan He Lin Chuang Bing Du Xue Za Zhi 1998 12 81

[B22] Wang LH Fu SH Tang Q Liang GD Construction of infectious chimeric cDNA clone of Sindbis virus by triple fusion PCR Bing Du Xue Bao 2006 22 107 113

[B23] Wang LH Fu SH Zhu WY Tang Q Liang GD Molecular basis of one-way serological Reaction between SINV and XJ-160 Virus Bing Du Xue Bao 2010 26 228 233 20572345

[B24] Klimstra WB Ryman KD Johnston RE Adaptation of Sindbis virus to BHK cells selects for use of heparan sulfate as an attachment receptor J Virol 1998 72 7357 7366 9696832 10.1128/jvi.72.9.7357-7366.1998 PMC109960

[B25] Byrnes AP Griffin DE Binding of Sindbis virus to cell surface heparan sulfate J Virol 1998 72 7349 7356 9696831 10.1128/jvi.72.9.7349-7356.1998 PMC109959

[B26] Heil ML Albee A Strauss JH Kuhn RJ An amino acid substitution in the coding region of the E2 glycoprotein adapts Ross River virus to utilize heparan sulfate as an attachment moiety J Virol 2001 75 6303 6309 10.1128/JVI.75.14.6303-6309.2001 11413296 PMC114352

[B27] Smit JM Waarts B-L Kimata K Klimstra WB Bittman R Wilschut J Adaptation of alphaviruses to heparan sulfate: interaction of Sindbis and Semliki forest viruses with liposomes containing lipid-conjugated heparin J Virol 2002 76 10128 10137 10.1128/JVI.76.20.10128-10137.2002 12239287 PMC136541

[B28] Zhu W Wang L Yang Y Jia J Fu S He Y Li JP Liang G Interaction of E2 glycoprotein with heparan sulfate is crucial for cellular infection of sindbis virus PLoS ONE 2010 5 3 e9656 10.1371/journal.pone.0009656 20300181 PMC2836379

[B29] Zhu WY Fu SH Li JP He Y Liang GD Amino acid substitutions in the E2 glycoprotein of Sindbis-like virus XJ-160 confer the ability to undergo heparan sulfate-dependent infection of mouse embryonic fibroblasts Virol J 2010 7 225 10.1186/1743-422X-7-225 20836893 PMC2944170

[B30] Xiong C Levis R Shen P Schlesinger S Rice CM Huang HV Sindbis virus: an efficient, broad host range vector for gene expression in animal cells Science 1989 243 1188 1191 10.1126/science.2922607 2922607

[B31] Yang YL Liang GD Fu SH Liang GD The construction of replicon of XJ-160 virus Bing Du Xue Bao 2003 18 221 226

[B32] Zhu WY Fu SH Wang LH Liang GD Construction of replicon vector derived from Sindbis virus Bing Du Xue Bao 2009 25 83 87 19678570

[B33] Zhu WY Li JJ Tang L Wang HQ Li J Fu JJ Liang GD Glycoprotein is enough for Sindbis virus-derived DNA vector to express heterogenous genes Virol J 2011 8 344 10.1186/1743-422X-8-344 21740598 PMC3148568

[B34] Zhu WY Liang GD Selection and characterization of packaging cell lines for XJ-160 Virus Intervirology 2009 52 100 109 10.1159/000215947 19420962

